# Brain network dysfunctions in addiction: a meta-analysis of resting-state functional connectivity

**DOI:** 10.1038/s41398-022-01792-6

**Published:** 2022-01-28

**Authors:** Serenella Tolomeo, Rongjun Yu

**Affiliations:** 1 Institute of High Performance Computing, Agency for Science, Technology and Research (A*STAR), Singapore, Singapore; 2 Department of Management, Hong Kong Baptist University, Hong Kong, China; 3 Department of Sport, Physical Education and Health, Hong Kong Baptist University, Hong Kong, China; 4 Department of Physics, Hong Kong Baptist University, Hong Kong, China

**Keywords:** Addiction, Physiology

## Abstract

Resting-state functional connectivity (rsFC) provides novel insights into variabilities in neural networks associated with the use of addictive drugs or with addictive behavioral repertoire. However, given the broad mix of inconsistent findings across studies, identifying specific consistent patterns of network abnormalities is warranted. Here we aimed at integrating rsFC abnormalities and systematically searching for large-scale functional brain networks in substance use disorder (SUD) and behavioral addictions (BA), through a coordinate-based meta-analysis of seed-based rsFC studies. A total of fifty-two studies are eligible in the meta-analysis, including 1911 SUD and BA patients and 1580 healthy controls. In addition, we performed multilevel kernel density analysis (MKDA) for the brain regions reliably involved in hyperconnectivity and hypoconnectivity in SUD and BA. Data from fifty-two studies showed that SUD was associated with putamen, caudate and middle frontal gyrus *hyperconnectivity* relative to healthy controls. Eight BA studies showed *hyperconnectivity* clusters within the putamen and medio-temporal lobe relative to healthy controls. Altered *connectivity* in salience or emotion-processing areas may be related to dysregulated affective and cognitive control-related networks, such as deficits in regulating elevated sensitivity to drug-related stimuli. These findings confirm that SUD and BA might be characterized by dysfunctions in specific brain networks, particularly those implicated in the core cognitive and affective functions. These findings might provide insight into the development of neural mechanistic biomarkers for SUD and BA.

## Introduction

Substance use disorder is characterized by excessive drug-seeking and taking [1]. Its core clinical symptoms comprise a chronically relapsing cycle of binging, intoxication, withdrawal and craving, despite the enormous adverse consequences. Behavioral addictions (BA) or non-substance addictions, such as gambling addiction, are defined as a set of behavior that the individual becomes dependent on. Both disorders are characterized by a persistent compulsion to seek and take a drug or perform a behavior, loss of control in limiting the intake, and they are often accompanied by negative emotions when the availability of the drug or behavior is prevented [2].

Both SUD and BA are complex multifaceted and multistage diseases. A previous rsfMRI systematic review reported that addictions would engage a range of brain networks, including the reward network, executive network and the habit and memory networks [3], and are broadly linked with changes in many cortical and subcortical brain regions. Among these addiction-related networks, it is crucial to identify key brain regions/networks that specifically contribute to addiction, in order to shed light on prevention and treatment. To assess the neural correlates of addictions, a number of functional neuroimaging studies have examined abnormalities in local brain regions and in communication between functionally distinct brain regions, as reported in two recent meta-analyses [4, 5]. These studies primarily used task-based functional magnetic resonance imaging (fMRI) to assess aberrant recruitment of brain regions in the context of different experimental paradigms and stages of addiction [6–9].

As an alternative approach to task-based fMRI, resting-state (rs) fMRI has been widely applied in both healthy participants and patients with neurological and psychiatric disorders [10–12]. Rs-fMRI is based on fluctuations of the blood-oxygenation-level-dependent (BOLD) signal, which characterize the intrinsic neuronal activity of the brain while subjects are in the awake state [13]. The literature evaluating rs-fMRI in substance and no-substance addiction is quite broad and includes seed-based functional connectivity (FC), regional homogeneity (ReHo), independent component analysis (ICA), amplitude of low-frequency oscillations (ALFF) and graph analysis under different types of addictions (for review see: Fedota and Stein, 2015; Ieong and Yuan, 2017, Pariyadath et al., 2016; Sutherland et al., 2012) [14–17].

Previously, Tahmasian and colleagues concluded that seed-based FC and effective connectivity are the standard methods to detect disruption of specific brain areas, whereas graph- and network-based analyses are valuable methods for assessing alterations across the whole brain networks [12]. These different methodological approaches have provided quite an ambiguous overview of the pathophysiological mechanisms underlying SUD and BA. For this reason, reviewing the literature, together with a quantitative meta-analysis, is needed to explain the inconsistencies between previously published works.

SUD and BA might have common disease aetiologies [18, 19]. It is important to highlight the similarities and differences between these two types of addictions. For example, similar to drug addiction [20, 21] a number of studies concluded that pathological gambling is characterized by white matter abnormalities [22–24] and reductions of cortical thickness [25]. However, one study reported increased corticolimbic connectivity in cocaine dependence, and a decrease in pathological gambling [19], suggesting that SUD and BA may also be associated with distinct brain abnormalities.

To the best of our knowledge, no previous rsFC studies have directly compared SUD and BA to probe for neural specificity. Individuals in general show unique patterns of addiction. Although a small proportion of addicts show addiction co-occurrence, many addicts struggle with one or more addictive behaviors but do not have difficulty with other types of addictive behaviors. For example, gambling addiction is only weakly associated with drug abuse. Addiction specificity describes this phenomenon: one addictive pattern may be acquired whereas another is not [26]. To date, the neurobiological evidence on why some addictions may not co-occur within the same individual has not been conclusively quantified. Similarly, the neural basis of co-occurrence of addictions remain elusive as well [27]. We believe that it is important to directly compare the neural substrates of various types of addiction and examine the neural specificity of each addiction. Such an approach may help explain addiction specificity and addiction co-occurence.

Here we conducted two coordinate-based meta-analysis approaches activation likelihood estimation (ALE) and Multilevel Kernel Density Analysis (MKDA). Both ALE and MKDA are coordinate-based meta-analysis (CBMA) approaches. Specifically, the ALE method involves the modeling of the reported loci of maximum activation as peaks of a 3D Gaussian probability, which is defined by a specified full-width half-maximum (FWHM). 3D Gaussian distribution produces a statistical map that assesses the likelihood of activation for each voxel as determined by all studies in the analysis [28]. Instead, the MKDA method establishes a binary map for each study, which are averaged giving the proportion of studies with any foci within a given radius from a voxel [29]. ALE focuses on the distribution of peak coordinates [28], while MKDA focuses on the distribution of statistical contrast maps [29]. We conducted a systematic review and three ALE meta-analyses of resting-state functional connectivity (rsFC) studies. Both hypo and hyper connectivities were examined delineate the abnormality patterns among intrinsic functional networks in substance use disorders and behavioral addictions. Finally, using the Multilevel Kerned Density Analysis (MKDA) meta-analytic technique, we aimed to replicate the findings of our first meta-analyses employed using a different meta-analytic technique [28].

## Methods

### Search strategy

**Fig. 1:**
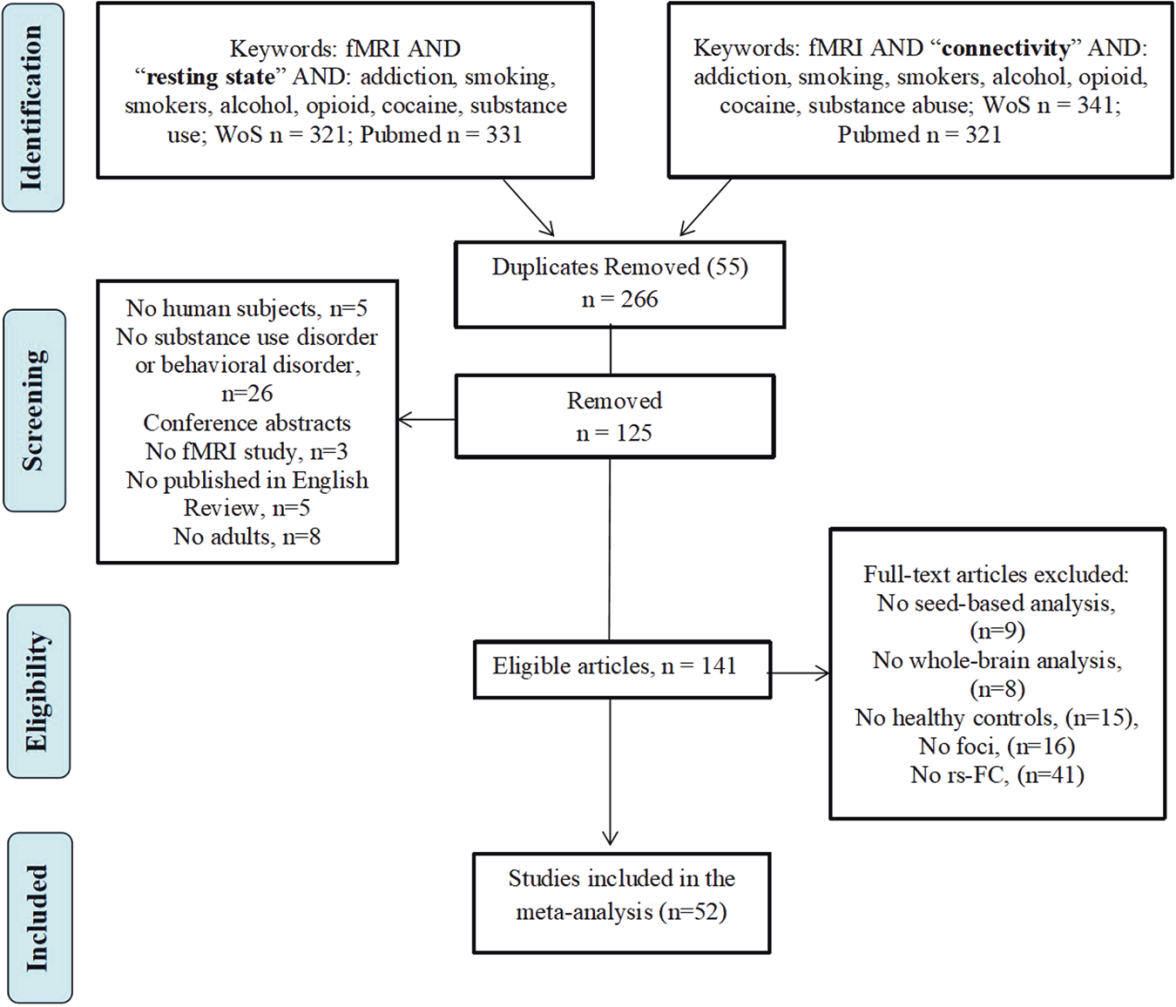
PRISMA flowchart. PRISMA flowchart for the selection of eligible studies.

A comprehensive literature search was carried out using Pubmed (https://www.ncbi.nlm.nih.gov/pubmed/) and Web of Science (http://www.webofknowledge.com) in August 2021. This was performed by combining a total of 18 searches using the key terms: [“rest” OR “resting”] and [“connect” OR “connectivity”], [“fMRI” OR “neuroimaging”] and various key terms corresponding to each search: “addiction”, “substance use disorder”, “substance abuse”, “alcohol”, “cocaine”, “opioid”, “smokers”, “smoking”, “heroin”, “stimulants”, “methamphetamine”, “gambling”, “gaming” and “internet gaming”. The search resulted in 139 papers and after screening seventy-two papers were reached. Figure 1 displays a PRISMA diagram of the specific search method reported. Notably, most resting-state connectivity meta-analysis analyses are based on these seventy-two studies, see Table 1 for further details.

**Table 1:** Demographic and clinical characteristics of SUD and BA studies included in the FC meta-analysis.

Article	HC (*n*)	Mean age, HC	SUD (*n*)	Mean age, SUD	Illness duration	Illness severity	SUD Phase	Substance
Camchong et al., 2013a [45]	23	47.99 (6.7)	23	48.46 (7.1)	–	–	Long-term abstinence	Alcohol
Camchong et al., 2013b [97]	23	47.99 (6.7)	36	47.85 (7.30)	–	–	Long-term abstinence	Alcohol
Halcomb et al., 2019 [98]			21 16	35.3 (11.4) 37.3 (11.6)	–	5.7 (3.5) 2.5 (0.8)	Users	Alcohol
Müller-Oehring et al., 2015 [99, 100]	26	49 (11)	27	50 (9)	–	AUDIT:26.6 (9.5); ACQ-R: 7.8 (2.6)	Abstinence	Alcohol
Wang et al., 2016 [101]	20	40.5 (8.2)	20	43.95 (6.3)	–	MAST: 31.50 (4.61)	Dependence	Alcohol
Wang et al., 2018 [102]	33	42.88 (6.05)	35	41.80 (9.53)	–	MAST: 33.46 (4.55)	Dependence	Alcohol
Weiland et al., 2014 [103]	87	25.8 (8.3)	255	31.1 (8.3)	–	AUDIT:14.9 (7.0)	Dependence	Alcohol
Liu et al., 2019	15	47.3 (4.9)	15	47.3 (5.0)	–	ADS: 21.5 (10.8) AUDIT: 17.1 (10.0)	Dependence	Alcohol
Blanco-Hinojo et al., 2017 [46]	29	22 (3)	28	21 (2)	6 years	–	Users	Cannabis
Pujol et al., 2014 [48]	28	22 (3)	27	21 (2)	–	–	Users	Cannabis
Zhou et al., 2018 [49]	28	23.39 (2.86)	24	24 (3.46)	–	–	Dependence	Cannabis
Adinoff et al., 2015 [50]	20	42.2 (8.9)	22	44.7 (6.4)	–	CCQ-Brief: 2.6 (9) OCCS: 23.9 (9.0)	Dependence	Cocaine
Contreras-Rodríguez et al., 2016 [19]	21	31 (4.6)	19	34.6 (6.8)	–	18.4 g/month	Dependence	Cocaine
Geng et al., 2017 [51]	67	39.99 (5.7)	64	40.59 (6.01)	4.24 years	–	Dependence	Cocaine
Gu et al., 2010 [52]	39	40 (5.1)	39	38 (6.2)	4.3 years	Use: $200/week	Dependence	Cocaine
Hu et al., 2015 [53]	56	38.7 (7.82)	56	39.86 (6.71)	12.64 years	Use: $246.70/week	Dependence	Cocaine
Kelly et al., 2011 [54]	24	35.1 (7.5)	25	35 (8.8)	11.43 years	CSSA: 12.48	Dependence	Cocaine
Martins et al., 2018 [55]	67	39.99 (5.7)	64	40.59 (6.01)	13 years	Use: $198	Using and dependence	Cocaine
McHugh et al., 2014 [56]	22	42.05 (8.4)	21	43.10 (6.84) 43.75 (7.53)	7.72 years 8.88 years	–	Dependence	Cocaine
McHugh et al., 2017 [57]	22	42.05 (8.4)	21	43.10 (6.84)	7.72 years	CCQ: 19.48 (10.73)	Dependence	Cocaine
Motzkin et al., 2014 [58]	18	31.7 (7.5)	22	32.0 (7.0)	–	ESI-SUB: 16	Dependence	Cocaine and other
Verdejo-Garcia et al., 2014 [59]	14	30.1 (8.8)	10	35.1 (8.9)	–	–	Dependence	Cocaine
Zhang and Li, 2018 [60]	66	39.3 (9.2)	66	41.4 (7.3)	20.2 years	CCQ: 23.8 (10.4)	Dependence	Cocaine
Li et al., 2013 [104]	15	31.9 (6.8)	14	35.4 (6.4)	7.44 years	0.6 g/day	Dependence	Heroin
Lin et al., 2018 [105]	30	41.47 (5.18)	30	42.44 (5)	7.66 years	52 mg/day	Dependence	Methadone
Wang et al., 2016 [68]	30	42.44 (5)	30	41.47 (5.18)	7.66 years	52 mg/day	Dependence	Heroin
Wang et al., 2016 [101]	30	38.9 (6.3)	30	40.7 (5.6)	14.4 years	2 g	Dependence	Heroin
Zhai et al., 2014 [69]	15	28.9 (8.12)	22	33.05 (6.04)	6.59 years	0.96 g/day	Dependence	Heroin
Zhang et al., 2015 [70]	15	27.79 (7.81)	21	33.07 (5.99)	6.20 years	0.85 g/day	Dependence	Heroin
Zou et al., 2015 [71]	29	38.9 (6.33)	30	40.73 (5.61)	14.40 years	–	Abstinence	Heroin
Kohno et al., 2014 [61]	27	33.8 (2.30)	25	35.68 (1.64)	8.59 years	3.57 g/week	Dependence	Methamphetamine
Kohno et al., 2016 [62]	18	38.9 (9.63)	20	37.0 (9.64)	6.82 years	–	Dependence	Methamphetamine
Kohno et al., 2018 [63]	20	33.4 (11.11)	30	37.62 (9.7)	12.04 years	–	Dependence	Methamphetamine
Li et al., 2020 [64]	31	34.48 (7.73)	34	32.15 (6.85)	6.59 years	–	Short-term Abstinence	Methamphetamine
Wang et al., 2019 [65]	21	29.52 (2.54)	16	28 (4.24)	–	–	Dependence	Amphetamine
Huang et al., 2014 [38]	10	22.5 (6.78)	11	23.7 (1.98)	–	FTND: 4.0	Dependence	Nicotine
Shen et al., 2017 [39]	41	39.46 (8.60)	84	38.23 (6.85)	20.70 years	FTND:5.23	Dependence	Nicotine
Shen et al., 2018 [40]	41	38.46 (8.60)	85	38.24 (6.81)	20.63 years	FTND: 5.18	Dependence	Nicotine
Um et al., 2019 [41]	62	35.31 (14.14)	34	34.15 (12.68)	16 years	FTND: 2.30	Users	Nicotine
Yuan et al., 2016 [43]	60	19.95 (1.8)	60	20.0 (1.7)	4.4 years	FTND: 6.0	Users	Nicotine
Zhang et al., 2017	37	32.81 (9.57)	37	33.11 (9.58)	15.05 years	FTND: 7	Users	Nicotine
Yu et al., 2017	27	19.5 (2.3)	27	19.4 (2.3)	–	FTND: 6.4	Dependence	Nicotine
Bi et al., 2017 [37]	40	19.8 (2.04)	40	19.62 (1.89)	4.20 years	FTND: 5,73	Dependence	Nicotine
Liu et al., 2016 [106, 107]	32	45.8 (9.3)	33	46.7 (9.4)	20.6 years	342 g/day	Dependence	Betel quid
Chen et al., 2016 [75]	30	24.14 (2.53)	30	23.64 (2.54)	–	CIAS: 83.14 (10.26)	Dependence	Internet gaming
Hong et al., 2015 [77]	11	14.81 (087)	12	13.41 (2.31)	–	YIAT: 57.00 (17.39)		Internet gaming
Lin et al., 2015 [78]	15	17.87	14	17.12 (2.73)	–	YIAS: 65.07 (13.25)	Dependence	Internet addiction
Yuan et al., 2017 [79]	44	19.5 (1.8)	43	19 (1.4)	–	IAT:61.2 (11.1)	Dependence	Internet gaming
Zhang et al., 2015 [80]	24	23.13 (2.09)	35	22.46 (2.21)	–	CIAS: 76.23 (7.67)	Dependence	Internet gaming
Zhang et al., 2016 [81]	41	23.02 (2.09)	74	22.28 (1.98)	7.28 years	CIAS: 78.36 (8.43)	Dependence	Internet gaming
Contreras-Rodríguez et al., 2016 [19]	21	31 (4.6)	19	33.8 (7.5)	–	40.8 h/month	Pathological	Gambling
Jung et al., 2014 [73]	15	26.60 (4.29)	15	27.93 (3.59)	2.20 years	PG-YBOCS: 16.13 (7.28)	Dependence	Gambling

Dependence = active users who are not abstinent; ACQ = Alcohol Craving Questionnaire; ADS = Alcohol Dependence Scale; CCQ-Brief = Cocaine Craving Questionnaire-Brief; CIAS = Chen Internet Addiction Scale; ESI-SUB = Externalizing Spectrum Inventory–Substance Abuse subscale; FTND = Fagerström Test for Nicotine Dependence; G-SAS = Gambling Symptom Assessment Scale; HC = healthy controls; IAT = Internet Addiction Test; MAST = Michigan Alcoholism Screening Test; *n* = sample size; OCCS = Obsessive-Compulsive Cocaine Scale; PG-YBOCS = Pathological Gambling Modification of Yale-Brown Obsessive Compulsive Scale; YIAS = Young’s Internet Addiction Scale; YIAST = Young’s Internet Addiction Test.

We included original functional magnetic resonance imaging (fMRI) studies that used seed-based rsFC:

1. To compare group differences in seed-based functional connectivity among SUD-HC were examined using the results of between-group contrasts (SUD < HC and SUD > HC).
2. To compare group differences in seed-based functional connectivity among BA-HC were examined using the results of between-group contrasts (BA < HC and BA > HC), respectively.

Effects were categorized based on the direction of effect (hyperconnectivity or hypoconnectivity in SUD or BA). Hyperconnectivity has been defined as larger positive or reduced negative rsFC and hypoconnectivity as larger negative or reduced positive rsFC compared with healthy controls.

### Study eligibility criteria

Studies focusing on other psychiatric comorbidities, such as depression, schizophrenia, anxiety, obsessive-compulsive disorder and neurological conditions were excluded as they have been separated in the DSM-V [30]. In the first screening of articles, the titles and abstracts were considered, and the following exclusion criteria were applied: (1) non-empirical studies, (2) non-human studies, (3) non-fMRI studies, (4) non-rsFC studies, (5) non-substance use disorder studies and (6) no adults. Subsequently, the full text of every article was further evaluated for eligibility. Studies were also excluded due to (1) not in English, (2) no HC group, (3) entries having the same seed regions of interest reported in another publication. These searches and exclusion criteria yielded a sample, *n* = 1911 for SUD + BA and *n* = 1580 for controls (Table 1). Coordinates were reported either in Talairach or Montreal Neurology Institute (MNI) coordinate space. The final dataset included seventy-two articles for SUD and BA > HC (310 contrasts) and seventy-two articles for the HC > SUD and BA (283 contrasts).

### Meta-analysis

GingerALE is a freely available, quantitative meta-analysis method developed by Turkeltaub et al [31] with the latest version described by Eickhoff and colleagues [28, 32] and Turkeltaub and colleagues [33]. Here, the latest version of GingerALE (3.0.2) was used (The BrainMap Database, www.brainmap.org; San Antonio, TX, USA), which relies on activation likelihood estimation (ALE) to compare coordinates compiled from multiple articles, estimate the magnitude of overlap, and yield clusters most statistically likely to become active across studies. The algorithm minimizes within-group effects and provides increased power by allowing for the inclusion of all relevant experiments [28, 33]. Talairach coordinates were converted to MNI with the Lancaster and colleagues (2007) transformation algorithm. Coordinates in MNI space were imported into the software. Imported foci were modeled using a full-width at half-maximum (FWHM) kernel estimated based on the corresponding experiment’s sample size as three-dimensional Gaussian spatial probability distributions [28, 33]. The resulting statistical maps were thresholded at *p* < 0.05 using a cluster-level correction for multiple comparisons and a cluster threshold at *p* < 0.05 [28]. Group differences were examined using contrast analyses. The threshold for group-contrasts was set to *p* < 0.05 uncorrected for multiple comparisons with 5000 permutations [34]. Group differences in resting-state functional connectivity were examined using the following six contrasts in ALE analyses: SUD + BA > HC and SUD + BA < HC; SUD > HC and SUD < HC; BA > HC and BA < HC; SUD > BA and SUD < BA.

### MKDA

Multilevel kernel density analysis (MKDA) was implemented through Matlab toolbox NeuroElf (http://neuroelf.net/) consistently with MKDA neuroimaging meta-analytic procedures [35, 36]. Contrast coordinates in Talaraich space were converted to MNI space. For all analyses, we used a priori threshold of *p* < 0.05 (family-wise error-corrected for multiple comparisons). Specifically, we investigated these meta-analytic contrasts as follow: [SUD > HC and SUD < HC] and [BA > HC and BA < HC].

## Results

### Included studies and sample characteristics

Meta-analyses were performed using GingerALE and consist of individuals with SUD (1911 subjects) and healthy controls (1580), both of which satisfied ALE power recommendations and include a minimum of 17 contrasts [28]. The mean age for SUD individuals and for healthy controls was 33.24 (±6.16) and 34.28 (±5.99) years old, respectively. See Supplementary Table S1 for more information regarding the rsFC methodology of the studies included in the meta-analyses.

For the SUD group, eight studies reported participants with nicotine addiction [37–44] and eight articles reported participants with alcoholism and/or harmful drinking habits [36–39, 42, 44, 45]. Four studies reported participants [46–49] who were cannabis users and 13 studies reported participants who were cocaine users [19, 50–60]. The remaining articles on SUD reported a variety of stimulants including methamphetamine/amphetamine (*n* = 5) [61–65], and heroin/methadone (*n* = 12) [66–71]. For the BA group, three articles reported participants with pathological gambling disorder [72–74] and eight studies reported participants with internet gaming disorder [66, 75–81].

### Meta-analytic resting-state functional connectivity

#### SUD results

A total of forty-four studies investigating rsFC abnormalities in SUD patients were identified. Table 3 shows a complete list of the independent meta-analysis of rsFC on SUD and HC only (ALE values are listed in Table 2).

**Table 2:** Results of the meta-analysis of resting-state functional connectivity in SUD and BA.

Cluster	Cluster size (mm^3^)	Brain regions	BA	*x*	*y*	*z*	ALE
***SUD*** > ***HC***							
1	18,160	L Putamen		−20	16	−6	0.0220
		L Caudate		−10	16	−2	0.0200
		L Middle Frontal Gyrus	44	−44	18	26	0.0200
		L Insula		−34	−6	20	0.0100
2	7200	R Putamen		26	6	4	0.0200
		R Globus Pallidus		16	8	2	0.0190
***BA*** > ***HC***							
1	20,592	R Putamen		26	6	−12	0.0110
		R Amygdala	21	48	−10	−12	0.0110
***SUD*** < ***HC***							
1	10,000	L Thalamus		−8	−14	2	0.0168
2	7312	R Medial Frontal Gyrus		4	44	38	0.0102
3	6432	R Parahippocampal Gyrus	28	28	−4	−20	0.0168
4	5152	L Insula	13	−42	12	10	0.0256
***BA*** < ***HC***							
1	14,416	R Medial Frontal Gyrus	9	22	46	18	0.0100
		R Anterior Cingulate	32	4	24	42	0.0090
2	12,328	L Thalamus		−6	−8	12	0.0090
		R Caudate		6	6	0	0.0090
***SUD*** ∩ ***BA***^***+***^							
1	2584	R Putamen		18	4	8	0.0096
***SUD*** > ***BA***^***+***^							
1	8112	L Claustrum		−30.2	17.2	4.1	0.1000
		L Caudate		−10.9	19.5	−5.6	0.1000
		L Putamen		−21.2	17	−9.2	0.0100
2	264	L Anterior Cingulate	24	−8	29	15	0.0100
***BA*** > ***SUD***^***+***^							
1	1544	R Putamen		24.4	2.4	−10	0.100
2	1400	R Insula		45	−9	−9	0.100
3	192	R Caudate		20.3	8.5	17.3	0.040
***SUD*** ∩ ***BA***^***−***^							
1	3168	R Medial Frontal Gyrus	6	0	36	32	0.0090
2	1904	L Thalamus		−24	−20	4	0.0090
***SUD*** < ***BA***^***−***^							
1	272	L Temporal Lobe	38	−48.9	4.1	−19.8	0.0400
***BA*** < ***SUD***^−^							
1	88	R Medial Frontal Gyrus	9	14.9	50.7	14.4	0.0500
2	24	R Anterior Cingulate	32	12	45.3	8.7	0.0500

MNI coordinates (*x*, *y*, *z*) of brain regions surviving a cluster-level threshold of *p* < 0.05 and a cluster forming threshold of *p* < 0.05 for single studies. Contrast threshold was set to *p* = 0.05, 5000 permutations, >50 mm^3^, ALE = Activation Likelihood Estimate; BA = Brodmann Area; BA = Behavioral Addiction; HC = Healthy Controls; ^***+***^ = Hyperconnectivity; ^***−***^ = Hypoconnectivity; SUD = Substance Use Disorders.

**Fig. 2:**
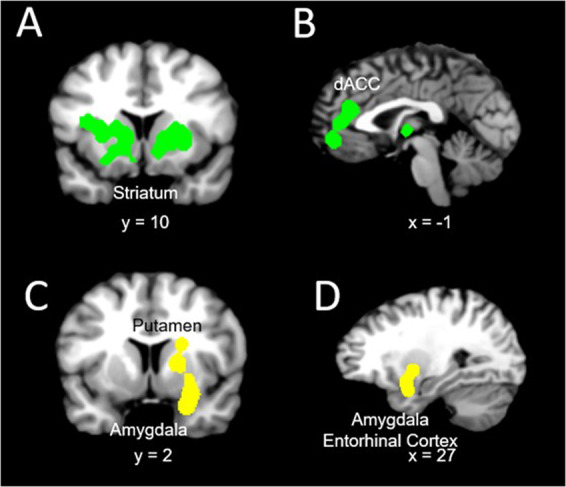
Concordant activation across SUD and BA. **A**, **B**: regions concordant across studies for SUD (in green) and **C**, **D**: regions concordant across studies for BA (in yellow).

The rsFC meta-analysis in SUD, when compared with HC, revealed the largest *hyperconnectivity* cluster to be within the striatum (putamen, caudate) and middle frontal gyrus (dorsolateral prefrontal cortex or DLPFC) (Fig. 2A and B). This was followed by a relatively large globus pallidus and anterior cingulate cluster and thalamus, as well as medial frontal gyrus (ventromedial prefrontal cortex or VMPFC) for the largest *hypoconnectivity* cluster (See Table 2).

#### BA Results

A total of eight studies investigating rsFC abnormalities in BA patients were identified. The majority of the identified studies investigated pathological gambling addiction and the remaining three studies investigated rsFC in internet gaming disorder. Table 2 shows a complete list of brain connectivity for independent meta-analyses on BA and HC (ALE values are listed in Table 2). The rsFC meta-analysis in BA included *hyperconnectivity* in regions within the putamen, amygdala, and medial frontal gyrus (Fig. 2C and D), and *hypoconnectivity* in the caudate, cingulate, and thalamus.

#### Conjunction and contrast analyses: SUD and BA Results

Conjunction and contrast analyses were performed to assess whether addiction specificity was present, based on previous reviews [26, 27]. While the conjunction analysis revealed *hyperconnectivity* for both SUD and BA in the putamen, the contrast analysis revealed *hyperconnectivity* in the claustrum, caudate, putamen and anterior cingulate for SUD (largest cluster size 8112) when compared with BA. The putamen/caudate and insula had the greatest *hyperconnectivity* cluster size in BA when compared with SUD (largest cluster size 1544). *Hypoconnectivity* for SUD and BA was shown in the medial frontal gyrus and thalamus. *Hypoconnectivity* within the temporal lobe was shown in SUD when compared with BA. The medial frontal gyrus and anterior cingulate revealed *hypoconnectivity* in BA when compared with SUD (ALE values are listed in Table 2).

#### SUD + BA Results

A total of seventy-two studies investigating rsFC abnormalities in SUD + BA were identified. Table 2 shows a complete list of brain activities for independent meta-analyses on SUD + BA and HC. Data from each cluster are listed in order of cluster size in MNI space identified by all ALE meta-analyses. Higher ALE values are indicative of a greater likelihood of rsFC (ALE values are listed in Table 3).

**Table 3:** Results of the meta-analysis of resting-state functional connectivity in SUD + BA and controls.

Cluster	Cluster size (mm^3^)	Brain regions	BA	*x*	*y*	*z*	ALE
***Healthy Control***^***+***^							
1	55,056	L Thalamus		−16	−10	12	0.0112
		R Midbrain		6	−12	−10	0.0103
2	21,672	L Cingulate	24	−8	34	10	0.0101
		R Medial Frontal Gyrus	32	6	32	44	0.0090
***Healthy Control***^***−***^							
1	11,640	L Parietal Lobe	7	−14	−60	60	0.0093
2	10,256	R Cerebellum		0	−76	−24	0.0092
***SUD*** + ***BA***^***+***^							
1	31,088	L Amygdala		−22	−6	−14	0.0159
		R Thalamus		10	−20	8	0.0141
		R Midbrain		10	−22	−8	0.0095
2	13,088	R Caudate		10	14	−4	0.0157
		R Putamen		30	−4	−10	0.0099
***SUD*** + ***BA***^***−***^							
1	6832	R Posterior Lobe		44	−66	−20	0.0090
2	5536	R Parahippocampal Gyrus		42	−32	−18	0.0090
***SUD*** + ***BA*** > ***Healthy Control***^***+***^							
1	20,864	L Caudate		−12	2	12	0.0209
		L Putamen		−20	16	−6	0.0193
		L Thalamus		−10	−16	14	0.0134
		L Insula	13	−34	12	14	0.0125
2	13,136	R Putamen		26	6	4	0.0244
		R Caudate Body		16	8	4	0.0222
		R Amygdala		26	−8	−28	0.0129
		R Parahippocampal Gyrus	34	16	−4	−18	0.0103
***SUD*** + ***BA*** < ***Healthy Control***^***−***^							
1	9832	R Dorsal Anterior Cingulate	32	8	36	22	0.0242
		R Dorso Medial Frontal Cortex	32	4	30	36	0.0219
		R Dorso Medial Frontal Cortex	6	2	24	44	0.0149

MNI coordinates (*x*, *y*, *z*) of brain regions surviving a cluster-level threshold of *p* < 0.05 and a cluster forming threshold of *p* < 0.05 for single studies. Contrast threshold was set to *p* = 0.05, 5000 permutations, >50 mm^3^, ALE = Activation Likelihood Estimate; BA = Brodmann Area; ^***+***^ = Hyperconnectivity; ^***−***^ = Hypoconnectivity.

**Fig. 3:**
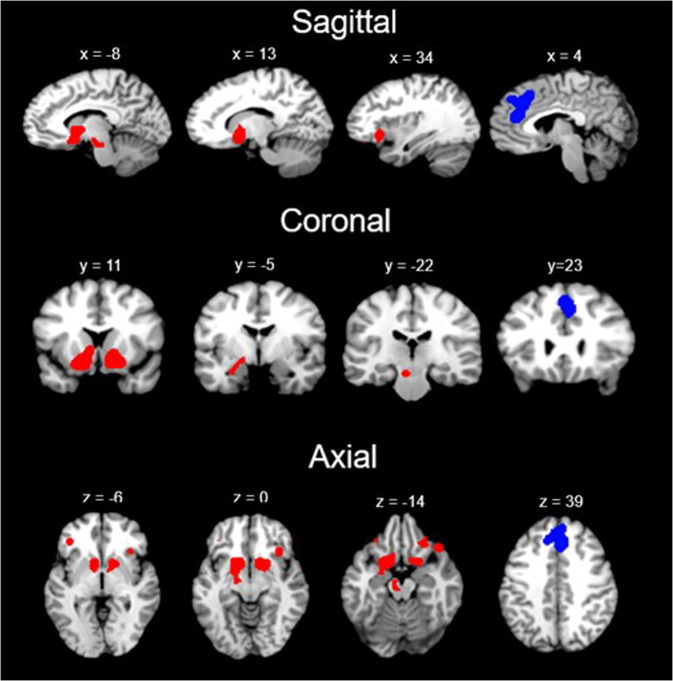
Concordant connectivity across SUD and BA. Concordant hyperconnectivity (red) and hypoconnectivity (blue) across SUD and BA (Sagittal, Coronal and Axial views).

**Fig. 4:**
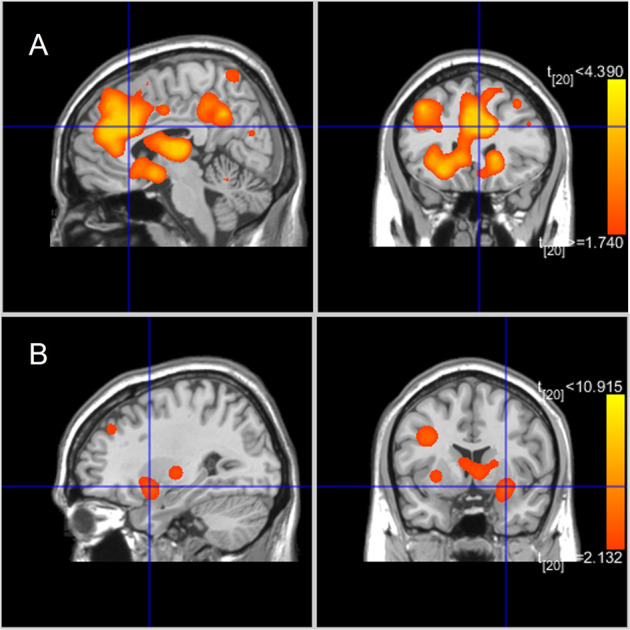
MKDA findings. Results from the MKDA analyses. **A** SUD vs controls and (**B**) BA vs controls.

For SUD + BA, the meta-analysis revealed that the largest *hyperconnectivity* cluster was within the amygdala, thalamus, and midbrain (Fig. 3) and relatively large parahippocampal gyrus, caudate, and putamen cluster, as well as *hypoconnectivity* in the posterior lobe and parahippocampal gyrus. For HC, the rsFC meta-analysis showed *hyperconnectivity* in the thalamus, midbrain, cingulate and frontal lobe and *hypoconnectivity* within the parietal lobe and cerebellum. The contrast analysis revealed *hyperconnectivity* in the basal ganglia, thalamus, insula, amygdala, and parahippocampal gyrus in SUD + BA in comparison with HC (See Table 3 for further details). MKDA results for SUD vs controls, and BA vs controls, are displayed in Fig. 4.

## Discussion

The need to better describe the human brain connectivity in SUD and BA has long been recognized, whereby meta-analyses serve as a crucial tool for consolidating evidence and streamlining the prevalent narrative (for a recent perspective see Suckling and Nestor, 2017; Yip et al., 2017) [18, 82]. To our knowledge, no previous meta-analysis has examined how connections between different brain areas during rest are altered in addictive neuropathologies. Here, we aimed to apply the ALE meta-analysis method to estimate convergence in functional connectivity rs-fMRI-based FC in substance use and behavioral disorders across studies.

We integrated findings from seventy-two rsFC studies and found convergent hyperconnectivity in individuals with SUD and/or BA: in the amygdala-basal ganglia, thalamus-midbrain and *hypoconnectivity* in the posterior lobe. In addition, basal ganglia, insula, amygdala and parahippocampal gyrus exhibited hyperconnectivity in the SUD group compared with the healthy control group. These findings show the enhancement of connectivity in the reward and salience networks, suggesting that altered physiology in the basal ganglia, midbrain, insula and medio-temporal lobe might be evaluated as specific biomarkers for drug addictions [3]. Interestingly, and importantly, our findings are consistent with an earlier systematic review of rsFC brain connectivity in drug addiction [17], which proposed that nicotine addiction had salience and executive network altered. This was confirmed by another recent review on chronic stimulant users which found enhanced coupling of reward, salience, and memory networks [83]. The hyperconnectivity of the reward and salience network in our meta-analysis might suggest that the recruited patients might be in a more chronic state of addiction [83]. The current meta-analysis expands upon previous results on specific brain regions by showing that abnormality in larger brain networks might be present in addiction.

Another novel finding of our ALE meta-analysis is the convergence of intrinsic functional patterns in the putamen in the SUD and BA, compared to HC. Numerous human and animal studies have identified putamen as the key region in SUD and HC, given its role in a variety of functions encompassing higher motor control, impulsivity and inhibitory control [77, 84–86]. In BA, a selective involvement of putamen functional connectivity in internet gaming disorder was revealed [77]. In addition, the putamen is part of the striatal “habit network” underlying learning of automatic behavior [19, 72].

Interestingly, no previous rsFC study has compared SUD and BA to probe the neural specificity. No studies have directly provide neural evidence to support the idea of addiction specificity [26, 27]. Here, we found that SUD showed *hyperconnectivity* in the basal ganglia (putamen, caudate, and globus pallidus), claustrum, middle frontal gyrus, and anterior cingulate compared with healthy controls. Instead, BA revealed *hyperconnectivity* in the putamen, temporal and frontal lobe. We suggest that the basal ganglia, claustrum and anterior cingulate are neuroanatomical substrates linked with SUD, which showed increased connectivity compared with BA. The importance of these regions in encoding rewards and/or reward-seeking and cognitive control has been demonstrated by functional imaging and human lesion studies [52, 87–90]. These increased connectivities for SUD adds further support to their central role specifically in the chronic phase of the addiction cycle. Much evidence exists on the capacity of drugs to enhance the mesolimbic dopamine system, whilst there is much less evidence in BA [52, 91–93].

Although our findings integrated a remarkably large sample size to establish consensus on the location of network disruptions in drug and behavioral addictions, limitations should be considered. First, the golden standard for directly detecting monosynaptic axonal pathways is the chemical tracer technique which requires ex-vivo tissue processing and can only be acquired in animal studies [94]. Thus, it is unclear how rsFC reflects the strength of monosynaptic and polysynaptic pathways. Second, the seventy-two experiments included in our meta-analysis differ in design, methodology, age, gender of the population, illness severity and duration of the use patterns (See Table 1 for further details). The wide variation of the substances used, and measures of quantity illustrate the need to report these factors more thoroughly and systematically in future studies. In addition, it highlights the need to standardize the reporting in future studies. Third, although the functional significance of positive and negative rsFC remains unclear, we lumped higher positive rsFC and lower negative rsFC in the SUD/BA vs. HC contrast, making it difficult to differentiate whether the differences were driven by higher rsFC in one group or lower rsFC in another group. The majority of studies only reported group differences without providing details about positive/negative rsFC in each group. Further studies may further explore the nature of group differences in rsFC.

Notably, there are several explanations for group differences in functional connectivity. It is possible that both groups may show positive functional connectivity and one group exhibits stronger positive functional connectivity than the other group. It is also possible that both groups show negative functional connectivity and one group exhibits weaker negative connectivity than the other group. The third possibility is that one group shows positive functional connectivity and the other group shows negative functional connectivity and hence there is a significant group difference. Unfortunately, in many of the original studies, the functional connectivity patterns in each group were not always reported. Hence, the current study cannot do separate within-group ALE analyses, e.g., one ALE analysis for positive connectivity in SUD group and one ALE analysis for negative connectivity in SUD group. We suggest that future empirical studies should aim to routinely report functional connectivity for each group before reporting group differences. Such practice might help researchers understand the nature of group differences and the pathophysiology of mental illnesses, including addictive disorders.

Fourth, due to the limited number of studies included we aggregated studies with heterogeneous patients, ranging from initial to abstinence stage, from short to long-term addictions. Analysis of subtypes of addictions and their cognitive functions and behavioral changes would be a strong supplement and would provide more context for each brain network. In the future, a meta-analysis with ReHo [95], ICA [96], ALFF and graph analysis studies is warranted.

In conclusion, the findings of this meta-analysis suggest that rsFC connectivity in drug and behavioral addictions are disrupted. Altered hyperconnectivity within salience or emotion processing may relate to deficits in regulating increased sensitivity to reward and salience stimuli. These findings might be helpful when attempting to identify potential putative markers for pharmacological interventions. A priority for future research would be to further identify how these unbalanced networks impact different phases (inclusive of intoxication, withdrawal and dependence) of the addiction cycle. These results might be used as indicatives of high risk in developing SUD or BA and might potentially guide effective treatments at an early stage.

## Supplementary information

Supplemental material
