# Supplementary material for: Brain network dysfunctions in addiction: a meta-analysis of resting-state functional connectivity
Source: Transl Psychiatry. 2022 Jan 28;12:41. doi: 10.1038/s41398-022-01792-6 (PMC8799706; doi:10.1038/s41398-022-01792-6)
Supplement: Supplementary file 1 — Supplemental material [file 41398_2022_1792_MOESM1_ESM.docx]

**Brain Network Dysfunction in Addiction: A Meta-analysis of Resting-State Functional Connectivity**

Serenella Tolomeo^1^, Rongjun Yu^2,3,4^

^1^ Institute of High Performance Computing, Agency for Science, Technology and Research (A*STAR), Singapore, Singapore

^2^ Department of Management, Hong Kong Baptist University, Hong Kong, China

^3^ Department of Sport, Physical Education and Health, Hong Kong Baptist University, Hong Kong, China

^4^ Department of Physics, Hong Kong Baptist University, Hong Kong, China

**SUPPLEMENTAL INFORMATION**

**Table S1.** Characteristics of participants.

| **Table S1.** Characteristics of participants | | | | | |  |  | |  |  |  |  |  |  |  |  |  |
| --- | --- | --- | --- | --- | --- | --- | --- | --- | --- | --- | --- | --- | --- | --- | --- | --- | --- |
|  | **Reference** | **Scanner** | **Tesla** | **Duration (Min)** | | | | **Software for Pre-Processing Data** | | **TR/TE (ms)** | | **EO/C** | **Flip Angle (degree)** | **Resolution (matrix size)** | **Seed Type** | **Region** | **Network** |
| 1 | Camchong et al., 2013a | Siemens Tim Trio | 3 | 4.06 | | | | FSL - AFNI | | 2000 | | C | 60 | 64*64 | Multiple Single Seeds | NAcc and sgACC | Reward |
| 2 | Camchong et al., 2013b | Siemens Tim Trio | 3 | 4.06 | | | | FSL - AFNI | | 2000 | | C | 60 | 64*64 | Multiple Single Seeds | NAcc and sgACC | Reward |
| 3 | Halcomb et al., 2019 | Siemens Prisma | 3 | 10 | | | | FSL | | 1200 | | C | 65 | 88*88 | Seed Regions | Insula | FPN |
| 4 | Müller-Oehring et al., 2015 | General Electric | 3 |  | | | | SPM8 | | 2200 | | O | 90 |  | Multiple Single Seeds | Seven Large-scale functional networks | DMN |
| 5 | Wang et al., 2016 | Siemens Trio | 3 |  | | | | SPM8 | | 2000 | |  | 90 | 64*64 | Multiple Single | Bilateral dACC, right dPCC, left mPFC, right OFC and left putamen | Reward |
| 6 | Wang et al., 2018 | Siemens Tim Trio | 3 |  | | | | SPM8  REST | | 2000 | | C | 90 | 64*64 | Multiple Single Seeds | Cuneus, precuneus, r dPFC, r PCC, bilateral thalamus | DMN |
| 7 | Weiland et al., 2014 | Siemens Trio | 3 | 5 | | | | SPM5 | |  | | O | 75 | 64*64 | Multiple Single Seeds | Executive control, basal ganglia and primary visual cortex | Reward |
| 8 | Liu et al., 2019 | Philips (Achieva) |  | 6min 10s | | | | AFNI, FSL | | 2000 | | C | 90 | 64*64 | Single Seeds | L and R Thalamus | Reward |
| 9 | Blanco-Hinojo et al., 2017 | General Electric Signa | 1.5 | | 6 | | | | SPM8 | | 2000 | C | 90 | 64*64 | Multiple Single Seeds | Basal ganglia and both internal (frontal cortex) and external (sensory cortices) sources of influence | Reward |
| 10 | Pujol et al., 2014 | Signa Excite system | 1.5 | | 6 | | | | SPM8 | | 2000 | C | 90 | 64*64 | Multiple Single Seeds | PCC and insula | DMN |
| 11 | Zhou et al., 2018 | Siemens TRIO | 3 | |  | | | | SPM12, FSL, AFNI | | 2580 | C | 80 | 64*64 | Multiple Single Seeds | Dorsal and ventral striatum | Reward |
| 12 | Adinoff et al., 2015 | Philips | 3 | | 6 | | | | AFNI | | 1525 | O | 70 |  | Multiple Single Seeds | L posterior hippocampus | DMN |
| 13 | Contreras-Rodríguez et al., 2016 | Philips Intera Achieva | 3 | | 6 | | | | SPM8 | | 2000 | C | 90 | 96*96 | Multiple Single Seeds | OFC, caudate, amygdala and thalamus | Reward |
| 14 | Geng et al., 2017 | Siemens Allegra | 3 | | 6 | | | | AFNI | |  | C | 70 | 64*64 | Multiple Single Seeds | Temporal pole and mPFC | DMN |
| 15 | Gu et al., 2010 | Siemens Allegra | 3 | | 6 | | | | AFNI – SPM 5 | | 2000 | C | 77 |  | Multiple Single Seeds | Six regions (VTA, NAcc, amygdala, hippocampus, thalamus and rACC) | Reward |
| 16 | Hu et al., 2015 |  | 3 | | 6 | | | |  | |  |  |  |  | Multiple Single Seeds | Striatal-cortical network | Reward |
| 17 | Kelly et al., 2011 | Siemens Allegra | 3 | | 6 | | | | AFNI - FSL | | 2000 | EO and C | 90 |  | Whole Brain and Multiple Seeds |  | DMN |
| 18 | Martins et al., 2018 | Philips Achieva | 1.5 | | 10.9 | | | | FSL 5.8 | | 3000 | C | 90 | 64*64 | Multiple Seeds | Default Mode Network | DMN |
| 19 | McHugh et al., 2014 | Philips | 3 | | 6 | | | | AFNI-FSL | | 1700 | O | 70 | 64*64 | Multiple Single Seeds | Amygdala (BLA and CMA) | Reward |
| 20 | McHugh et al., 2017 | Philips | 3 | | 6 | | | | AFNI-FSL | | 1700 | O | 70 | 64*64 | Whole Brain | DMN, SN and ECN networks | DMN |
| 21 | Motzkin et al., 2014 | Mind Research Network’s Siemens | 1.5 | |  | | | |  | | 2000 |  | 75 | 64*64 | Multiple Single | ACC | FPN |
| 22 | Verdejo-Garcia et al., 2014 | General Electric Signa Excite | 1.5 | | 4 | | | | SPM | | 2000 | C | 90 | 64*64 | Multiple Single | ACC, PAG and insula | FPN |
| 23 | Zhang and Li, 2018 | Siemens Trio | 3 | | 10 | | | | SPM8 | | 2000 | C | 85 | 64*64 | Single Seeds | Ventral Striatum | Reward |
| 24 | Li et al., 2013 | General Electric Signa | 3 | |  | | | | SPM5 | |  |  |  | 64*64 |  | PCC | DMN |
| 25 | Lin et al., 2018 | General Electric Signa | 3 | |  | | | | SPM8 | | 2000 | C |  | 64*64 | Multiple Single Seeds | dPFC | DMN |
| 26 | Wang et al., 2016 | Siemens Trio | 3 | | 8 | | | | SPM8 | | 2000 | C | 90 | 64*64 | Multiple Single Seeds | Area surrounding parietal and occipital cortex | DMN |
| 27 | Wang et al., 2016 | General Electric Signa | 3 | | 8 | | | | REST | |  |  |  | 64*64 | Single Seeds | Insula | FPN |
| 28 | Zhai et al., 2014 | General Electric Signa |  | | 6 | | | | AFNI – SPM8 | | 2000 | C | 90 | 64*64 | Multiple Single Seeds | Hippocampus | DMN |
| 29 | Zhang et al., 2015 | General Electric Signa | 3 | |  | | | | AFNI – SPM8 | | 2000 |  | 90 | 64*64 | Multiple Single Seeds | dACC, rACC and sgACC | FPN |
| 30 | Zou et al., 2015 | Siemens Magnetom Trio | 3 | | 8 | | | | SPM8 | | 2000 | C |  | 64*64 | Single Seed | NAcc | Reward |
| 31 | Kohno et al., 2014 | Siemens Magnetom Trio | 3 | |  | | | | FSL | |  |  | 90 | 64*64 | Multiple Single Seeds | Midbrain- r dPFC | DMN |
| 32 | Kohno et al., 2016 | Siemens Trio |  | |  | | | | FSL | |  |  | 90 | 64*64 | Multiple Single Seeds | Midbrain | Reward |
| 33 | Kohno et al., 2018 | Siemens Tim Trio | 3 | |  | | | | FSL | | 2000 | C | 80 | 128*128 | Multiple Single Seeds | Striatum and dPFC | Reward |
| 34 | Li et al., 2020 | Siemens Trio | 3 | | 8 | | | | SPM12 REST | | 2000 | C | 62 |  | Multiple Single Seeds | Cerebellar-cerebral networks | DMN |
| 35 | Wang et al., 2019 |  | 1.5 | |  | | | | DPARSF | | 3000 | C | 90 | 64*64 | Single Seed | NAcc | Reward |
| 36 | Huang et al., 2014 | Philips | 3 | | 7 min 5 s | | | | DPARSF | | 1500 |  | 80 |  | Multiple Single Seeds | ACC and DMN regions | DMN |
| 37 | Shen et al., 2017 | General Electric Signa | 3 | | 6 min 16 s | | | | SPM8 | | 2000 | C | 80 | 64*64 | Single Seed | Amygdala | Reward |
| 38 | Shen et al., 2018 | General Electric Signa | 3 | |  | | | | DPARSF | | 2000 | C | 80 | 64*64 | Single Seeds | Cerebellum | DMN |
| 39 | Um et al., 2019 | Siemens Magnetom Trio Tim | 3 | | 5 | | | | AFNI | | 2500 |  | 80 | 72*72 | Multiple Single Seeds | Bilateral NAcc, insula and amydala | Reward |
| 40 | Yuan et al., 2016 | EXCITE, General Electric | 3 | | 6 min 10 s | | | | AFNI | | 2000 | C | 90 | 64*64 | Multiple Single Seeds | Bilateral caudate, putamen, NAcc | Reward |
| 41 | Zhang et al., 2017 |  | 3 | | 10 | | | |  | |  |  |  |  | Multiple Single Seeds | BNM, VS | Reward |
| 42 | Yu et al., 2018 | Philips | 3 | |  | | | | FMRIB | | 2000 | C | 90 | 64*64 | Single Seed | Prefrontal cortex | DMN |
| 43 | Bi et al., 2017 | EXCITE, General Electric | 3 | | 6 min 10 s | | | | AFNI-FSL | | 2000 | C | 90 | 64*64 | Multiple Single Seeds | L and R insula (posterior and anterior) | FPN |
| 44 | Liu et al., 2016 | Siemens Verio | 3 | |  | | | | SPM8 DPARSF | | 2000 | C | 80 | 64*64 | Single Seeds | Bilateral ACC | FPN |
| 45 | Chen et al., 2016 | General Electric Signa | 3 | | 10 min 33 s | | | | REST | |  | C |  | 64*64 | 64*64 | Insula, NAcc | Reward |
| 46 | Hong et al., 2015 | Siemens Magnetom Trio Trim | 3 | | 6 min 45 s | | | | SMP8 | | 2700 | C | 90 | 64*64 | Multiple Single Seeds | Caudate nucleus and putamen | Reward |
| 47 | Lin et al., 2015 | Tesla Philips Achieva | 3 | |  | | | | SMP8 | | 2000 | C | 90 | 64*64 | Multiple Single Seeds | Caudate and putamen | Reward |
| 48 | Yuan et al., 2017 | EXCITE, General Electric | 3 | | 6 min 10 s | | | | AFNI-FSL | | 2000 | C | 90 | 64*64 | Multiple Single Seeds | Bilateral caudate, putamen and NAcc | Reward |
| 49 | Zhang et al., 2015 | Siemens Tim Trio | 3 | |  | | | | DPARSF | | 2000 | O | 90 | 64*64 | Multiple Single Seeds | R NAcc and VTA | Reward |
| 50 | Zhang et al., 2016 | Siemens Tim Trio | 3 | |  | | | | SMP8 | | 2000 | O | 90 | 64*64 | Multiple Single Seeds | L, R ventral insula and LF posterior insula | Reward |
| 51 | Contreraras Rodriguez et al., 2016 | Intera Achieva Philips | 3 | | 6 | | | | SPM8 | | 2000 | C | 90 | 96*96 | Multiple Single Seeds | Corticostriatal network involving OFC, caudate, thalamus and amygdala | Reward |
| 52 | Jung et al., 2014 | Siemens (Avanto system) | 1.5 | |  | | | | FSL | | 11.6 | C | 90 |  | Single Seed | DMN, PCC | Reward |

*Note:* studies varied greatly in methods such as type of scanner, duration of scan, repetition time/echo time (TR/TE), instructing participants to keep eyes open (O) or closed (C)). BA=Broadman Area; BNM=Basal nucleus of Meynert; dACC= dorsal anterior cingulate cortex; DMN=Default Mode Network; DPARSF= Data Processing Assistant for Resting-state fMRI dPFC=dorsolateral Prefrontal Cortex; min= minutes; NAcc=Nucleus Accumbens; r=right; PCC= Posterior Cingulate Cortex; rACC= rostral anterior cingulate cortex; sgACC=subgenual anterior cingulate cortex; VTA= Ventral Tegmental area; VS=Ventral Striatum.

**References**

1 Camchong J, Stenger A, Fein G. Resting-State Synchrony in Long-Term Abstinent Alcoholics. *Alcohol Clin Exp Res* 2013; **37**: 75–85.

2 Camchong J, Stenger VA, Fein G. Resting state synchrony in long-term abstinent alcoholics with versus without comorbid drug dependence. *Drug Alcohol Depend* 2013; **131**: 56–65.

3 Halcomb ME, Chumin EJ, Goñi J, Dzemidzic M, Yoder KK. Aberrations of anterior insular cortex functional connectivity in nontreatment-seeking alcoholics. *Psychiatry Res - Neuroimaging* 2019; **284**: 21–28.

4 Müller-Oehring E, Jung Y, … AP-C, 2015 undefined. The resting brain of alcoholics. *academic.oup.com*https://academic.oup.com/cercor/article-abstract/25/11/4155/2366345 (accessed 29 May2020).

5 Wang J, Fan Y, Dong Y, Ma M, Ma Y, Dong Y *et al.* Alterations in brain structure and functional connectivity in alcohol dependent patients and possible association with impulsivity. *PLoS One* 2016; **11**. doi:10.1371/journal.pone.0161956.

6 Wang J, Fan Y, Dong Y, Ma M, Dong Y, Niu Y *et al.* Combining gray matter volume in the cuneus and the cuneus-prefrontal connectivity may predict early relapse in abstinent alcohol-dependent patients. *PLoS One* 2018; **13**. doi:10.1371/journal.pone.0196860.

7 Weiland BJ, Sabbineni A, Calhoun VD, Welsh RC, Bryan AD, Jung RE *et al.* Reduced left executive control network functional connectivity is associated with alcohol use disorders. *Alcohol Clin Exp Res* 2014; **38**: 2445–2453.

8 Liu J, Cai W, Zhao M, Cai W, Sui F, Hou W *et al.* Reduced resting‐state functional connectivity and sleep impairment in abstinent male alcohol‐dependent patients. *Hum Brain Mapp* 2019; **40**: 4941–4951.

9 Blanco-Hinojo L, Pujol J, Harrison BJ, Macià D, Batalla A, Nogué S *et al.* Attenuated frontal and sensory inputs to the basal ganglia in cannabis users. *Addict Biol* 2017; **22**: 1036–1047.

10 Pujol J, Blanco-Hinojo L, Batalla A, López-Solà M, Harrison BJ, Soriano-Mas C *et al.* Functional connectivity alterations in brain networks relevant to self-awareness in chronic cannabis users. *J Psychiatr Res* 2014; **51**: 68–78.

11 Zhou F, Zimmermann K, Xin F, Scheele D, Dau W, Banger M *et al.* Shifted balance of dorsal versus ventral striatal communication with frontal reward and regulatory regions in cannabis-dependent males. *Hum Brain Mapp* 2018; **39**: 5062–5073.

12 Adinoff B, Gu H, Merrick C, McHugh M, Jeon-Slaughter H, Lu H *et al.* Basal hippocampal activity and its functional connectivity predicts cocaine relapse. *Biol Psychiatry* 2015; **78**: 496–504.

13 Contreras-Rodríguez O, Albein-Urios N, Vilar-López R, Perales JC, Martínez-Gonzalez JM, Fernández-Serrano MJ *et al.* Increased corticolimbic connectivity in cocaine dependence versus pathological gambling is associated with drug severity and emotion-related impulsivity. *Addict Biol* 2016; **21**: 709–718.

14 Geng X, Hu Y, Gu H, Salmeron B, Adinoff B, Brain ES- *et al.* Salience and default mode network dysregulation in chronic cocaine users predict treatment outcome. *academic.oup.com*https://academic.oup.com/brain/article-abstract/140/5/1513/3038013 (accessed 1 Jun2020).

15 Gu H, Salmeron BJ, Ross TJ, Geng X, Zhan W, Stein EA *et al.* Mesocorticolimbic circuits are impaired in chronic cocaine users as demonstrated by resting-state functional connectivity. *Neuroimage* 2010; **53**: 593–601.

16 Hu Y, Salmeron BJ, Gu H, Stein EA, Yang Y. Impaired functional connectivity within and between frontostriatal circuits and its association with compulsive drug use and trait impulsivity in cocaine addiction. *JAMA Psychiatry* 2015; **72**: 584–592.

17 Kelly C, Zuo XN, Gotimer K, Cox CL, Lynch L, Brock D *et al.* Reduced interhemispheric resting state functional connectivity in cocaine addiction. *Biol Psychiatry* 2011; **69**: 684–692.

18 Martins DLN, Valiatti TD de S, D’Ávila J, Ferreira LF, Batista EK, Bazán PR *et al.* A conectividade funcional extrínseca da rede de modo padrão em usuários de crack-cocaína. *Radiol Bras* 2018; **51**: 1–7.

19 McHugh MJ, Demers CH, Salmeron BJ, Devous MD, Stein EA, Adinoff B. Cortico-amygdala coupling as a marker of early relapse risk in cocaine-addicted individuals. *Front Psychiatry* 2014; **5**. doi:10.3389/fpsyt.2014.00016.

20 McHugh MJ, Gu H, Yang Y, Adinoff B, Stein EA. Executive control network connectivity strength protects against relapse to cocaine use. *Addict Biol* 2017; **22**: 1790–1801.

21 Motzkin JC, Baskin-Sommers A, Newman JP, Kiehl KA, Koenigs M. Neural correlates of substance abuse: Reduced functional connectivity between areas underlying reward and cognitive control. *Hum Brain Mapp* 2014; **35**: 4282–4292.

22 Verdejo-Garcia A, Contreras-Rodríguez O, Fonseca F, Cuenca A, Soriano-Mas C, Rodriguez J *et al.* Functional alteration in frontolimbic systems relevant to moral judgment in cocaine-dependent subjects. *Addict Biol* 2014; **19**: 272–281.

23 Zhang S, Li CSR. Ventral striatal dysfunction in cocaine dependence - Difference mapping for subregional resting state functional connectivity. *Transl Psychiatry* 2018; **8**. doi:10.1038/s41398-018-0164-0.

24 Li Q, Yang WC, Wang YR, Huang YF, Li W, Zhu J *et al.* Abnormal function of the posterior cingulate cortex in heroin addicted users during resting-state and drug-cue stimulation task. *Chin Med J (Engl)* 2013; **126**: 734–739.

25 Lin HC, Wang PW, Wu HC, Ko CH, Yang YH, Yen CF. Altered gray matter volume and disrupted functional connectivity of dorsolateral prefrontal cortex in men with heroin dependence. *Psychiatry Clin Neurosci* 2018; **72**: 435–444.

26 Wang L, Zou F, Zhai T, Lei Y, Tan S, Jin X *et al.* Abnormal gray matter volume and resting-state functional connectivity in former heroin-dependent individuals abstinent for multiple years. *Addict Biol* 2016; **21**: 646–656.

27 Zhai TY, Shao YC, Xie CM, Ye EM, Zou F, Fu LP *et al.* Altered intrinsic hippocmapus declarative memory network and its association with impulsivity in abstinent heroin dependent subjects. *Behav Brain Res* 2014; **272**: 209–217.

28 Zhang Y, Gong J, Xie C, Ye EM, Jin X, Song H *et al.* Alterations in brain connectivity in three sub-regions of the anterior cingulate cortex in heroin-dependent individuals: Evidence from resting state fMRI. *Neuroscience* 2015; **284**: 998–1010.

29 Zou F, Wu X, Zhai T, Lei Y, Shao Y, Jin X *et al.* Abnormal resting-state functional connectivity of the nucleus accumbens in multi-year abstinent heroin addicts. *J Neurosci Res* 2015; **93**: 1693–1702.

30 Kohno M, Morales AM, Ghahremani DG, Hellemann G, London ED. Risky decision making, prefrontal cortex, and mesocorticolimbic functional connectivity in methamphetamine dependence. *JAMA Psychiatry* 2014; **71**: 812–820.

31 Kohno M, Okita K, Morales AM, Robertson CL, Dean AC, Ghahremani DG *et al.* Midbrain functional connectivity and ventral striatal dopamine D2-type receptors: Link to impulsivity in methamphetamine users. *Mol Psychiatry* 2016; **21**: 1554–1560.

32 Kohno M, Loftis JM, Huckans M, Dennis LE, McCready H, Hoffman WF. The relationship between interleukin-6 and functional connectivity in methamphetamine users. *Neurosci Lett* 2018; **677**: 49–54.

33 Li X, Su H, Zhong N, Chen T, Du J, Xiao K *et al.* Aberrant Resting-State Cerebellar-Cerebral Functional Connectivity in Methamphetamine-Dependent Individuals After Six Months Abstinence. *Front Psychiatry* 2020; **11**: 191.

34 Wang Y, Yan KJ, Fan CX, Luo XN, Zhou Y. Altered functional connectivity of the nucleus accumbens subdivisions in amphetamine-type stimulant abusers: A resting-state fMRI study. *BMC Neurosci* 2019; **20**: 66.

35 Huang W, King JA, Ursprung WWS, Zheng S, Zhang N, Kennedy DN *et al.* The development and expression of physical nicotine dependence corresponds to structural and functional alterations in the anterior cingulate-precuneus pathway. *Brain Behav* 2014; **4**: 408–417.

36 Shen Z, Huang P, Wang C, Qian W, Luo X, Guan X *et al.* Altered function but not structure of the amygdala in nicotine-dependent individuals. *Neuropsychologia* 2017; **107**: 102–107.

37 Shen Z, Huang P, Wang C, Qian W, Yang Y, Zhang M. Cerebellar Gray Matter Reductions Associate With Decreased Functional Connectivity in Nicotine-Dependent Individuals. *Nicotine Tob Res* 2018; **20**: 440.

38 Um M, Hummer TA, Cyders MA. Relationship of negative urgency to cingulo-insular and cortico-striatal resting state functional connectivity in tobacco use. *Brain Imaging Behav* 2019; : 1–12.

39 Yuan K, Yu D, Bi Y, Li Y, Guan Y, Liu J *et al.* The implication of frontostriatal circuits in young smokers: A resting-state study. *Hum Brain Mapp* 2016; **37**: 2013–2026.

40 Sheng Z, Sien H, Lisa M F, Xingguang L, Carolyn M M, Laszlo Z *et al.* Resting-State Functional Connectivity of the Basal Nucleus of Meynert in Cigarette Smokers: Dependence Level and Gender Differences. *Nicotine Tob Res* 2017; **19**. doi:10.1093/NTR/NTW209.

41 Yu D, Yuan K, Bi Y, Luo L, Zhai J, Liu B *et al.* Altered interhemispheric resting-state functional connectivity in young male smokers. *Addict Biol* 2018; **23**: 772–780.

42 Bi Y, Yuan K, Guan Y, Cheng J, Zhang Y, Li Y *et al.* Altered resting state functional connectivity of anterior insula in young smokers. *Brain Imaging Behav* 2017; **11**: 155–165.

43 Liu T, Li J, Zhao Z, Zhong Y, Zhang Z, Xu Q *et al.* Betel quid dependence is associated with functional connectivity changes of the anterior cingulate cortex: A resting-state fMRI study. *J Transl Med* 2016; **14**: 1–13.

44 Chen C-Y, Yen J-Y, Wang P-W, Liu G-C, Yen C-F, Ko C-H. Altered Functional Connectivity of the Insula and Nucleus Accumbens in Internet Gaming Disorder: A Resting State fMRI Study. *Eur Addict Res* 2016; **22**: 192–200.

45 Hong SB, Harrison BJ, Dandash O, Choi EJ, Kim SC, Kim HH *et al.* A selective involvement of putamen functional connectivity in youth with internet gaming disorder. *Brain Res* 2015; **1602**: 85–95.

46 Lin F, Zhou Y, Du Y, Zhao Z, Qin L, Xu J *et al.* Aberrant corticostriatal functional circuits in adolescents with internet addiction disorder. *Front Hum Neurosci* 2015; **9**: 356.

47 Yuan K, Yu D, Cai C, Feng D, Li Y, Bi Y *et al.* Frontostriatal circuits, resting state functional connectivity and cognitive control in internet gaming disorder. *Addict Biol* 2017; **22**: 813–822.

48 Zhang JT, Ma SS, Yip SW, Wang LJ, Chen C, Yan CG *et al.* Decreased functional connectivity between ventral tegmental area and nucleus accumbens in Internet gaming disorder: Evidence from resting state functional magnetic resonance imaging. *Behav Brain Funct* 2015; **11**: 37.

49 Zhang JT, Yao YW, Li CSR, Zang YF, Shen ZJ, Liu L *et al.* Altered resting-state functional connectivity of the insula in young adults with Internet gaming disorder. *Addict Biol* 2016; **21**: 743–751.

50 Jung MH, Kim JH, Shin YC, Jung WH, Jang JH, Choi JS *et al.* Decreased connectivity of the default mode network in pathological gambling: A resting state functional MRI study. *Neurosci Lett* 2014; **583**: 120–125.
